# Optimizing Dizziness Management: Impact of Audiology Triage on Care Efficiency

**DOI:** 10.1002/lary.70305

**Published:** 2025-12-06

**Authors:** Sanjena Venkatesh, Daniel S. Talian, Kim Le, Archer R. Schaeffer, Keshav Shah, Tanvi Garneni, Christian Jung, Sherrie Davis, Michael J. Ruckenstein, Douglas C. Bigelow, Tiffany P. Hwa

**Affiliations:** ^1^ Perelman School of Medicine at the University of Pennsylvania Philadelphia Pennsylvania USA; ^2^ Department of Otorhinolaryngology – Head and Neck Surgery University of Pennsylvania Philadelphia Pennsylvania USA; ^3^ Department of Audiology University of Pennsylvania Philadelphia Pennsylvania USA; ^4^ University of Pennsylvania Philadelphia Pennsylvania USA

**Keywords:** audiology, dizziness, referral patterns, vestibular dysfunction, wait times

## Abstract

**Objectives:**

To evaluate the impact of a vestibular audiology‐driven triage system on care efficiency for patients presenting with dizziness.

**Methods:**

We conducted a retrospective review of all patients who underwent comprehensive Balance Function Testing (BFT) at the University of Pennsylvania vestibular clinic between September 2019 and June 2023. The new vestibular triage model was implemented in early 2021. Data collection comprised patient demographics, initial audiology diagnoses, referral destinations, final medical diagnoses, and time to specialty visit. Linear regression models were employed to assess temporal trends in referral patterns and wait times.

**Results:**

Three‐thousand three hundred and five patients (mean age 55.9 ± 16.5 years; 34.6% male) were included for analysis. Referrals to neurotology significantly decreased over time (*p* < 0.0001), while neurology referrals significantly increased (*p* < 0.0001); ENT referrals showed a non‐significant decline (*p* = 0.14). Time from BFT to neurology (*p* = 0.0012) evaluation significantly decreased over the course of the study period, while neurotology (*p* = 0.18) and ENT (*p* = 0.16) wait times showed non‐significant downward trends.

**Conclusion:**

Vestibular audiology‐driven triage represents a promising approach for managing patients with dizziness, resulting in significant changes to referral patterns and potentially improving the efficiency of specialty resource allocation. Future research should explore patient‐centered outcomes to further assess its clinical impact.

**Level of Evidence:**

4.

## 1 Introduction

Dizziness affects approximately 30% of the adult population, ranking as the third most common overall outpatient complaint and the leading complaint among patients over the age of 75 [1, 2, 3]. Given its nonspecific nature and broad differential encompassing central neurologic disorders, cardiogenic conditions, psychiatric manifestations, and peripheral vestibular dysfunctions, diagnosis is often challenging [1, 4, 5]. The diverse potential etiologies span multiple medical specialties (i.e., neurotology, general otolaryngology, neurology, cardiology, ophthalmology, and primary care), making it difficult to determine the appropriate specialty for care [6, 7]. Though dizziness is typically benign, a small subset of cases may signal more sinister pathology, such as cerebrovascular disease or neoplastic processes. Prompt referral and diagnosis are thus crucial, both to address potential serious pathology and to improve quality of life in benign cases.

Research has increasingly highlighted the role vestibular audiologists can play in the assessment and triage of dizzy patients. Studies have demonstrated audiologists' proficiency in conducting and interpreting balance function tests (BFTs) with high accuracy, suggesting that leveraging this expertise could improve care efficiency [8, 9, 10, 11].

**FIGURE 1:**
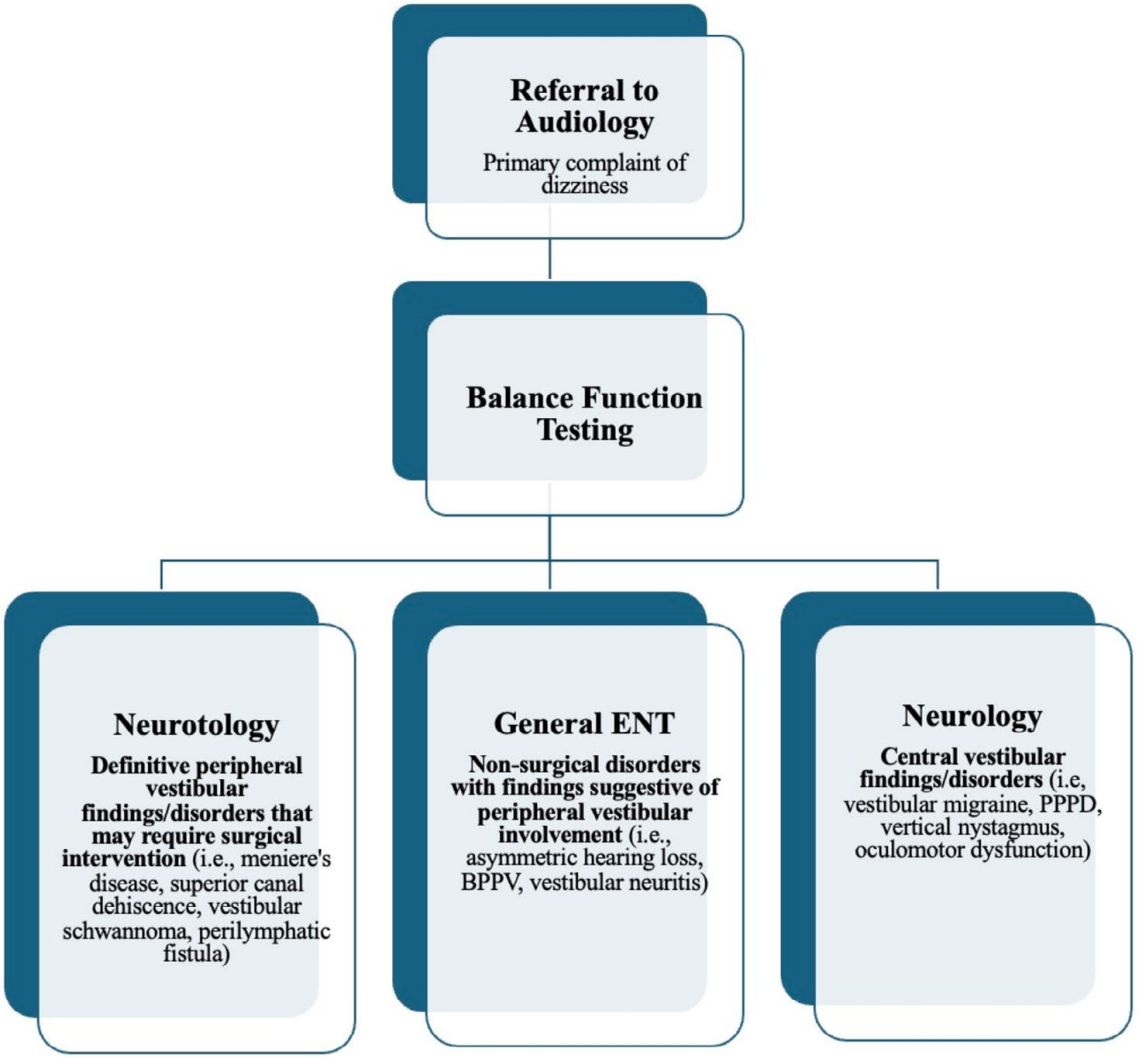
Triage algorithm. [Color figure can be viewed in the online issue, which is available at www.laryngoscope.com]

At our institution, we are fortunate to have a comprehensive balance center that can be leveraged for triage. Historically, the majority of patients who underwent Balance Function Testing (BFTs) with audiology for vestibular dysfunction were referred or otherwise requested for neurotology referral at the time of balance center evaluation, irrespective of the balance test findings. This approach resulted in inefficient resource utilization, as most patients presenting with dizziness did not require specialized neurotologic intervention. Patients were thus often redirected between multiple specialists, leading to prolonged diagnostic journeys and delays in appropriate treatment. Recognizing this issue, a new triage algorithm was introduced in early 2021. Rather than defaulting most patients to neurotology, vestibular audiologists began formally triaging patients to the most appropriate specialty based on their BFT results and clinical presentation. Crucial to this model is a clear delineation of specialty roles. At our center, neurotologists focus exclusively on peripheral vestibular conditions that may require operative management; they do not manage patients with central vestibular disorders. General ENTs manage non‐surgical disorders with findings suggestive of peripheral vestibular involvement. Neurologists evaluate patients with suspected central findings, as well as conditions such as vestibular migraine and persistent postural‐perceptual dizziness. The scope of each specialty likely varies across institutions, but this division forms the foundation of our triage protocol. A schematic of the triage algorithm is shown in Figure 1.

The present study evaluates the impact of this new triage model at our institution. The primary objectives are to analyze changes in audiology‐initiated specialty referral frequency and time to specialty visit over the course of this shift. Secondary objectives include assessing diagnostic accuracy of vestibular audiology, as well as patient compliance with referrals. All in all, this study seeks to determine the effectiveness of vestibular audiology in triage, with the goal of reducing unnecessary specialist visits, minimizing time to specialty visit, and ultimately improving patient care for those suffering from dizziness.

## 2 Methods

### 2.1 Study Design

This study was approved by the Institutional Review Board of the University of Pennsylvania Health System. A retrospective review was conducted of electronic medical records for all patients who underwent BFT at the Hospital of University of Pennsylvania (HUP) multidisciplinary Balance and Dizziness Center between September 2019 and June 2023. The new vestibular triage model was formally implemented in early 2021, though its adoption and full integration occurred gradually over several months. Patients were included if they presented with a primary complaint of dizziness and underwent comprehensive BFTs. A comprehensive BFT here includes video‐nystagmography, computerized dynamic posturography, rotational chair testing, and video head impulse testing. Exclusion criteria included patients presenting for BFTs for audiologic testing, cochlear implant candidacy evaluation, or those with incomplete medical records.

Data collection comprised patient demographics, audiology diagnosis, audiology referral destination, final diagnosis, and time to specialist visit. Audiology diagnosis was defined as the initial diagnosis assigned by the audiologist following BFT. Audiology referral destination indicated the specialty to which the audiologist referred the patient. Final diagnosis was determined by the treating medical provider and considered stable if unchanged for at least 12 months. Finally, time to specialist visit was recorded for patients who received a new referral, capturing the interval between BFT and the first appointment with the referred specialist. These times were recorded for visits included within Care Everywhere, a system which allows sharing of electronic healthcare information across select healthcare organizations in Epic.

General descriptive statistics were conducted to characterize the cohort as a whole. Primary outcomes—referral frequencies and average time to visit—were tabulated by month over the course of the study period. Temporal trends in referral patterns and wait times were assessed via linear regression models, with two‐tailed t tests performed to evaluate whether the slopes differed significantly from zero. Statistical significance was defined as *p* < 0.05, and 95% confidence intervals (CI) were constructed where appropriate. Secondary outcomes included diagnostic accuracy and compliance rates. The accuracy of audiology diagnoses was assessed by calculating the percentage of cases in which the audiologist's suggested diagnosis matched the final diagnosis determined by the medical provider. Compliance rates were calculated as the proportion of patients who attended a visit with the specialty to which they were referred. Data analysis was performed using R (version 4.1.2, Foundation for Statistical Computing, Vienna, Austria) and Prism (version 10.3.0, GraphPad Software, San Diego, CA, USA).

## 3 Results

A total of 3305 patients underwent comprehensive BFTs at HUP between September 2019 and June 2023 and were included for analysis. The cohort comprised 34.5% males (*n* = 1144), with a mean age of 55.9 (±16.5) years. Complete descriptive characteristics are highlighted in Table 1.

**TABLE 1:** Sample characteristics.

Variable	Value
*n*	3305
Age (mean)	55.9 (±16.5)
Male (*n*, %)	1144 (34.5%)
Audiology referrals (*n*, %)	
Neurotology	1125 (34.0%)
ENT	1032 (31.2%)
Neurology	985 (29.8%)
Final diagnosis (*n*, %)	
Migraine	652 (19.7%)
BPPV	417 (12.6%)
Meniere's disease	289 (8.7%)
PPPD	125 (3.8%)
Vestibular neuritis/labyrinthitis	100 (3.0%)
Other	159 (4.8%)
Unspecified dizziness	805 (24.3%)
No final diagnosis (lost to follow‐up)	758 (22.9%)

### 3.1 Referral Trends and Wait Times

**FIGURE 2:**
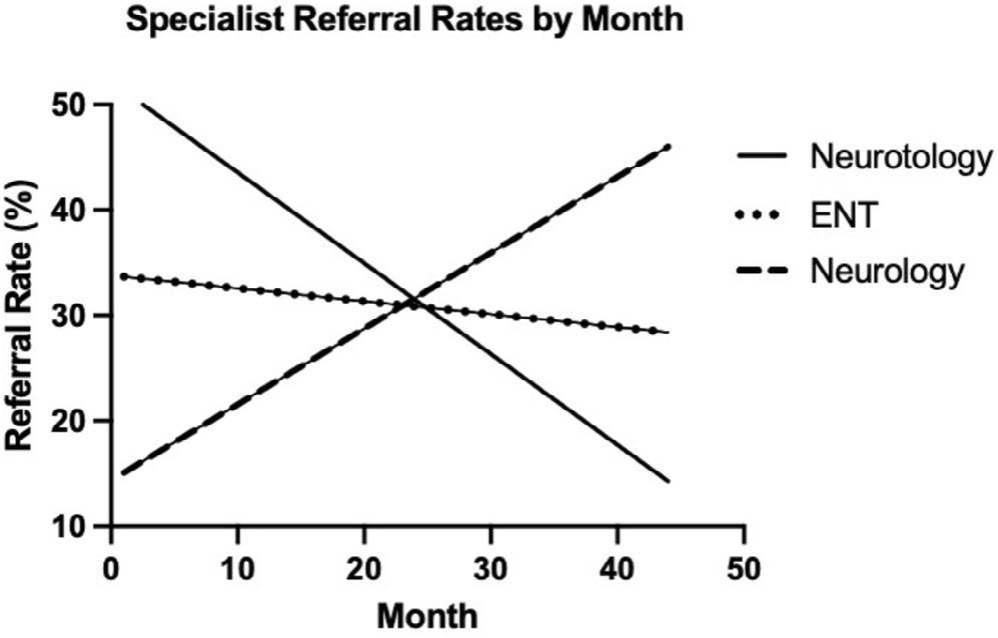
Referral rates from the balance center to neurotology, ENT, and neurology by month from September 2019 to June 2023. This demonstrates decreases in neurotology and ENT referrals, with a corresponding increase in neurology referrals over the course of the study period.

Balance center referral rates were trended over time (Figure 2). Neurotology referrals demonstrated a significant decrease over the course of the study period (*β* = −0.86 [−1.09, −0.63], *p* < 0.0001). In the first 6 months of the study, an average of 62.4% of patients (range: 33.3%–67.1%) were referred to neurotology, which declined to 18.6% (range: 10.4%–25.5%) by the final 6 months. ENT referrals showed a non‐significant decline (*β* = −0.12 [−0.29, 0.04], *p* = 0.14). Conversely, neurology referrals exhibited a significant upward trend (*β* = 0.72 [0.51, 0.92], *p* = < 0.0001), increasing from an initial six‐month average of 5.0% (range: 0.0%–8.2%) to 37.1% (range: 27.3%–43.3%) by the final 6 months. A subset of patients received additional referrals following the initial audiologist‐directed referral; these results are summarized in Table 2. Overall, however, the total number of specialist referrals decreased from an average of 1.21 in the first 6 months to 0.74 in the last 6 months.

**TABLE 2:** Subsequent specialty referrals following initial referral.

Original referral	Subsequent referral to neurotology (%)	Subsequent referral to ENT (%)	Subsequent referral to neurology (%)
Neurotology	—	10.5	22.4
ENT	16.2	—	25.0
Neurology	15.4	21.6	—

**FIGURE 3:**
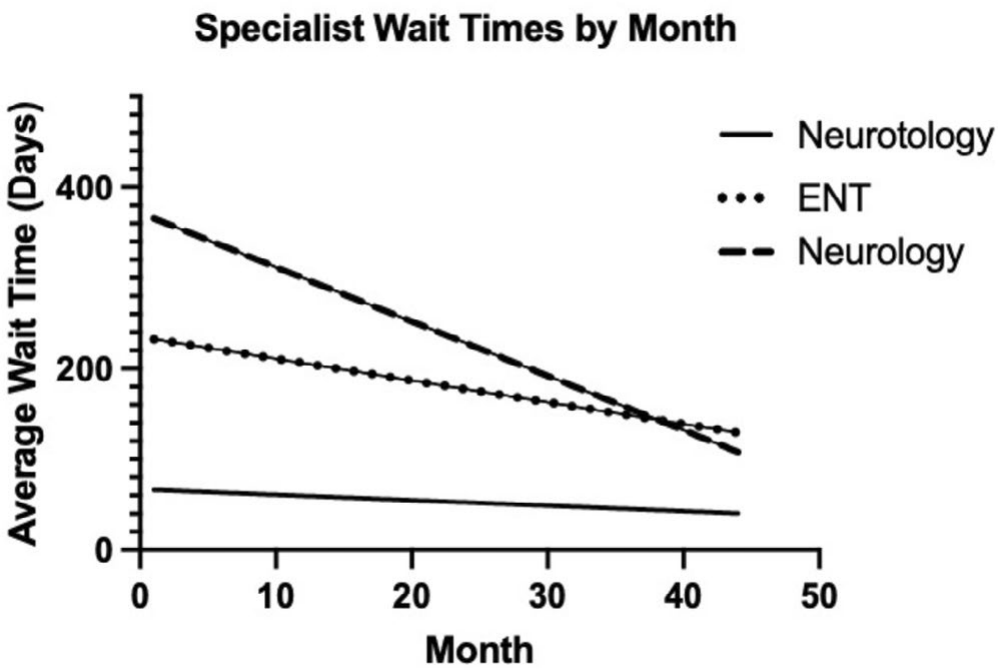
Wait times for new neurotology, ENT, and neurology referrals by month from September 2019 to June 2023. This demonstrates decreases in wait times across specialties over the course of the study period.

Average times from new referral to specialty visit were analyzed across the three departments (Figure 3). Neurotology and ENT wait times showed non‐significant decreasing trends (*β* = −0.60 [−1.48, 0.28], *p* = 0.18; *β* = −2.4 [−5.79, 0.99], *p* = 0.16). Neurology wait times, in contrast, exhibited a significant decreasing trend (*β* = −5.99 [−9.47, −2.50], *p* = 0.0012).

### 3.2 Diagnosis Alignment

A significant portion of cases (22.9%) were lost to follow‐up, with no provider seen after balance testing, resulting in no final diagnosis. An additional 24.3% saw a provider but received no definitive diagnosis, with dizziness attributed to an unspecified etiology. Otherwise, the most common diagnosis was vestibular migraine (19.7%), followed by benign paroxysmal positional vertigo (BPPV) (12.6%), Meniere's disease (8.7%), persistent postural‐perceptual dizziness (PPPD) (3.8%), and vestibular neuritis/labyrinthitis (3.0%). 4.8% of patients received “other” diagnoses (i.e., post‐concussive syndrome, long COVID, etc.). Among cases without missing diagnoses, the suggested diagnosis corresponded with the final medical diagnosis in 98.4% of cases for Meniere's disease, 73.7% for vestibular migraine, 73.4% for BPPV, 46.2% for vestibular neuritis/labyrinthitis, and 45.0% for PPPD. Table 3 summarizes the patterns of diagnostic discrepancy between audiology impressions and final MD diagnoses.

**TABLE 3:** Patterns of diagnostic discrepancy between audiologist impression and physician diagnosis.

Audiologist impression	Concordance rate	Most common discordant diagnosis, *n* (%)	Second most common discordant diagnosis, *n* (%)
BPPV (*n* = 349)	73.4%	Migraine, 65 (18.9%)	Meniere's, 16 (4.6%)
Migraine (*n* = 498)	73.7%	BPPV, 72 (14.5%)	PPPD, 30 (6.0%)
Meniere's (*n* = 63)	98.4%	Migraine, 6 (9.5%)	BPPV, 3 (4.8%)
Vestibular neuritis (*n* = 13)	46.2%	BPPV, 3 (23.1%)	Migraine, 3 (23.1%)
PPPD (*n* = 20)	45.0%	Migraine, 6 (30%)	BPPV, 2 (10%)

*Note*: In several cases, providers documented multifactorial dizziness with multiple contributing diagnoses, which explains why percentages may exceed 100%.

### 3.3 Patient Compliance

Across the neurotology, ENT, and neurology referrals, overall compliance was 65.0%. By specialty, compliance was highest for neurotology at 90.1%, followed by ENT at 55.3% and neurology at 46.9%.

## 4 Discussion

This study aimed to evaluate the impact of a vestibular audiology‐driven triage system on referral patterns and wait times for patients presenting with dizziness. To our knowledge, this represents the first institutional analysis of vestibular audiologist‐driven triage, offering valuable insights into an innovative approach to managing patients with dizziness.

### 4.1 Referral Trends and Wait Times

Our primary finding highlights a significant shift in referral patterns following the implementation of this triage system. We observed a decline in neurotology and ENT referrals over the study period, with a corresponding increase in neurology referrals. Stratifying by final diagnosis, we found that referral patterns largely aligned appropriately with specialty expertise. For example, vestibular migraine and PPPD cases were most commonly referred to neurology (57.9% and 45.2%, respectively), while Meniere's disease was predominantly routed to neurotology (91.1%). This suggests that vestibular audiologists may effectively be able to direct patients to the appropriate providers.

Wait times showed decreasing trends across specialties. Neurotology and ENT wait times showed non‐significant decreases. Though not significant, this supports the idea that a more selective triage process may alleviate some of the capacity burden on these specialties, with more efficient resource use and fewer inappropriate referrals. Neurology experienced a significant decrease in wait times despite a rise in referral volumes. This somewhat unexpected pattern may be due to neurology's larger provider base or greater scheduling flexibility, which could allow the department to absorb increased patient load without proportionate delays. It is also likely that external institutional factors (i.e., changes in staffing or scheduling protocols) played a role. Taken together, these results suggest that targeted triage may contribute to more efficient access to care.

Our findings of reduced referrals and more efficient care are consistent with the broader literature on vestibular triage. Kasbekar et al. similarly reported decreased rates of secondary referrals via audiology‐led triage for dizziness, with the majority of patients being treated and discharged after their first visit [10]. Riska et al. also demonstrated improved care efficiency through the implementation of a BPPV‐focused audiology triage clinic, showing a 23‐day reduction in wait times [11]. These findings carry important clinical and systems‐level implications. For patients, earlier connection to the most appropriate specialist may shorten the time to definitive diagnosis and treatment, minimizing the frustration and delays in care that often accompany repeated evaluations by multiple providers. At the systems level, this approach offers a promising strategy to optimize allocation of specialty resources, allowing for reduction of unnecessary healthcare utilization. Given that dizziness accounts for close to $50 billion in annual healthcare expenditures, any intervention that can improve efficiency and reduce redundant care has the potential for meaningful cost savings [12, 13]. Vestibular audiology‐driven triage thus offers promise for enhancing both patient outcomes and healthcare system performance.

### 4.2 Diagnosis Alignment

We observed varying alignment rates between audiologists' suggested diagnoses and final medical provider diagnoses across different vestibular conditions. High corroboration was achieved for Meniere's disease, vestibular migraine, and BPPV, suggesting vestibular audiologists can reliably identify these conditions. However, lower alignment rates were observed for vestibular neuritis/labyrinthitis and PPPD.

These patterns highlight the value of audiologists providing diagnostic impressions while acknowledging their expected limitations. Audiologists, after all, are not expected to diagnose all causes of dizziness with complete accuracy. BFTs are limited—they offer objective physiologic data that can rule in or out peripheral vestibular dysfunction but aren't designed to assess broader neurologic, psychological, or systemic causes of dizziness. Audiologists enhance the care pathway by offering informed diagnostic impressions based on available test data, with the understanding that final diagnosis and management decisions for certain conditions appropriately require physician evaluation.

### 4.3 Patient Compliance

Patient compliance with referrals varied substantially by specialty, with the highest compliance seen for neurotology, followed by ENT and neurology. It is important to note that our study does not account for visits outside of the Care Everywhere system; actual compliance rates may be higher if patients sought care from external providers.

The disparity in compliance likely reflects patient buy‐in regarding the perceived relevance of each specialty to their symptoms. For example, patients referred to neurotology—a subspecialty explicitly focused on vestibular disorders—may perceive these specialists as having more targeted expertise for dizziness compared to general ENT physicians or neurologists, leading to higher follow‐through. Practical factors may also contribute to these disparities, including ease of scheduling, insurance coverage, and wait times across specialties. In our tertiary care center, the neurotology and ENT clinics are physically connected to the balance center, facilitating a smoother referral process with clearer pathways for appointment scheduling. Neurology referrals, in contrast, may present greater logistical challenges. Especially within our system, neurology appointments are known to have long wait times, which could in turn discourage follow‐through.

It is also important to recognize that not all patients require medical follow‐up after vestibular testing. Dizziness symptoms are often self‐limiting. After discussing diagnoses such as BPPV or PPPD, for example, many patients may feel reassured that there is no life‐threatening cause for their symptoms and choose not to pursue further specialty care. In these cases, referrals are typically offered as an option, rather than a strongly recommended course of action, leading some patients to opt out of follow‐up.

### 4.4 Alternative Approaches

While the vestibular audiology‐driven triage model shows promise, it is important to note that this approach is unique to our tertiary care institution, which boasts a comprehensive balance center staffed with trained vestibular audiologists. This may not be applicable, and in turn, replicable in all clinical settings. Alternative triage methods can also be effective, and their utility may depend on the available resources and clinical context. In the current era, machine learning has emerged as a promising tool for triaging dizzy patients. Several studies have demonstrated the potential of artificial intelligence models for symptom triage with relative success [14, 15, 16, 17]. For instance, Romero‐Brufau et al. developed a triage algorithm that yielded comparable results to clinicians in directing dizzy patients to appropriate specialists [17]. Other research has validated the utility of specialized surveys in triage [18, 19, 20, 21, 22, 23]. These studies highlight the effectiveness of pre‐encounter history questionnaires in categorizing vestibular diagnoses and guiding patients to the appropriate specialty care. Ultimately, various options exist, and it is crucial to select an approach that best aligns with the resources, workflows, and needs of a particular clinical setting.

### 4.5 Strengths, Limitations, and Future Directions

Our study offers several important strengths that distinguish it from prior research. Firstly, our study is the largest to date, encompassing a substantial cohort of over 3300 patients and spanning multiple years. The large sample size and extended follow‐up period allow for a more meaningful analysis of trends in referral behavior and wait times over time. In addition, our study stands out in its rigorous design by tracking outcomes across multiple specialties (ENT, neurotology, and neurology). This multi‐specialty perspective enriches our understanding of how audiology triage impacts different types of care pathways. Finally, we go beyond referral rates to evaluate additional key outcomes such as time to specialist evaluation, diagnostic alignment between audiology and medical providers, and patient follow‐through with recommended referrals. This holistic evaluation offers a deeper understanding of not only where patients are being referred, but how efficiently they are progressing through the care pathway after triage. Thus, our study meaningfully extends the literature on the subject.

Several limitations must be acknowledged, however. First, as a single‐center retrospective study, our findings may not be generalizable to all healthcare settings, particularly in community hospitals or primary care practices that lack dedicated balance centers. Second, our study focused primarily on process metrics rather than patient‐centered outcomes, such as symptom resolution, quality of life improvements, or patient satisfaction with care. Although we observed a decrease in referrals and wait times, this does not necessarily translate to improved care if patients are not receiving the appropriate treatment. Without outcome measures, we cannot definitively conclude that our triage approach has enhanced overall patient care quality. Additionally, the audiologist‐driven approach employed in our study is resource‐intensive and potentially costly when compared to alternative triage methods, such as standardized questionnaires or machine learning algorithms. This raises important questions about its cost‐effectiveness and scalability.

Future research should address the limitations of the current study by implementing comprehensive, prospective designs with longer follow‐up periods. While our analysis of referral trends provides valuable insights into resource utilization, subsequent studies must incorporate patient‐centered outcome measures to assess whether audiologist‐driven triage meaningfully improves symptom resolution, quality of life, and satisfaction with care. These metrics are essential for determining the clinical value of this approach beyond its impact on referral efficiency. Research comparing different triage models—including audiologist‐driven assessment, standardized questionnaires, machine learning algorithms, and primary care‐based approaches—would also help identify optimal strategies for different healthcare settings and patient populations. Such comparative studies should include formal cost‐effectiveness analyses that consider both direct costs (personnel, equipment, facilities) and indirect costs (time to diagnosis, unnecessary testing, patient travel burden) to determine the most efficient approach to vestibular triage. Overall, the goal is to streamline care and meaningfully improve outcomes for patients with dizziness.

## 5 Conclusion

Vestibular audiology‐driven triage represents a promising approach for managing patients with dizziness, resulting in significant changes to referral patterns and potentially improving the efficiency of specialty resource allocation. By redirecting patients to more appropriate specialties earlier in their care journey, this model addresses inefficiencies in the management of vestibular disorders. While our single‐center experience may not be universally applicable due to resource requirements, it provides valuable insights into one effective approach to vestibular triage. Future research should incorporate patient‐centered outcome measures, compare various triage models, and seek to address the concerning proportion of dizzy patients who remain undiagnosed. Ultimately, optimizing the care pathway for patients with dizziness requires continued innovation in both diagnostic approaches and clinical workflows to ensure timely, appropriate care for this common yet challenging presentation.

## Funding

The authors have nothing to report.

## Conflicts of Interest

The authors declare no conflicts of interest.

## Data Availability

The data that support the findings of this study are available on request from the corresponding author. The data are not publicly available due to privacy or ethical restrictions.
